# Epidemiology of chronic hypoparathyroidism in Italy: a systematic review and meta-analysis

**DOI:** 10.1007/s40618-026-02827-1

**Published:** 2026-02-05

**Authors:** T. C. M. Topini, P. Stagni, M. Dobreva, C. Galeone, C. Cipriani, S. Pirri, Maria Domenica Sanna

**Affiliations:** 1 Ascendis Pharma Bone Diseases, Hellerup, Denmark; 2 Regulatory Pharma Net srl, Pisa, Italy; 3 Bicocca-Applied Statistics Center (B-ASC), Università degli Studi di Milano Bicocca, Milan, Italy; 4 Department of Medical and Cardiovascular Sciences, Sapienza University of Rome, Rome, Italy

**Keywords:** HPT, Epidemiology, Italy, Incidence, Prevalence

## Abstract

**Purpose:**

Hypoparathyroidism (HPT) is an endocrine disease caused by insufficient levels of parathyroid hormone (PTH). PTH is the primary regulator of the calcium/phosphate balance, acting directly on bone and kidney, and indirectly on the intestine. Regarding the epidemiology of chronic HPT, there are limited data in Italy. This study aimed to establish incidence and prevalence of chronic HPT in Italy.

**Methods:**

A systematic literature review was conducted to identify the current knowledge regarding the epidemiology of chronic HPT in Italy, and a random effect meta-analysis was performed to estimate the pooled incidence. A subgroup analysis of non-surgical and postsurgical HPT estimates for patients was also conducted.

**Results:**

We identified 4 studies eligible for inclusion reporting data from 15,412 patients with HPT. Considering a thyroidectomy rate of 57 per 100,000 and the incidence of post-surgical chronic HPT (≥ 6 months after surgery) at 1.2% (95% CI: 0.7%-1.8%), the number of incident post-surgical chronic HPT was estimated in 353 cases. Based on a mean time with chronic HPT after diagnosis of 17.4 years, a total of 6,142 prevalent post-surgical chronic HPT patients has been estimated for Italy. Findings from this analysis showed 4,383 prevalent non-surgical chronic HPT cases in Italy. The prevalence of chronic HPT, both surgical and non-surgical, in the adult population in Italy has been estimated to be 10,524 cases.

**Conclusion:**

This study provides a current estimate of the prevalence and incidence of chronic HPT in Italy. In this analysis, postsurgical chronic HPT accounted for the majority of prevalent cases. Epidemiological studies with appropriate study design are necessary to provide a validated estimation of the prevalence and incidence of HPT in Italy.

## Introduction

Hypoparathyroidism (HPT) is a rare endocrine disease caused by insufficient levels of parathyroid hormone (PTH), a primary regulator of calcium and phosphate balance [1–3]. Biochemically, the diagnosis of HPT is made when serum PTH levels are lower insufficient in the presence of hypocalcemia. Hyperphosphatemia and elevated urinary calcium excretion, particularly in individuals receiving conventional therapy (active vitamin D and calcium), are other hallmarks of the disease [2, 4, 5].

Clinical presentation of HPT includes neuromuscular irritability, cardiovascular and renal disease, a low bone turnover state and high bone mineral density, cognitive impairment, and reduced health-related quality of life (QoL) [1–3, 6–9].

Approximately 75% of cases of HPT are a consequence of the unintended removal or damage of the parathyroid glands during neck surgery; non-surgical HPT may have autoimmune, genetic, or metabolic etiology [3, 10]. Earlier guidelines, including the 2016 Endocrine Society Guidelines, the 2019 Canadian and International Consensus Statement, and the 2022 European Society of Endocrinology Consensus Statement, defined chronic HPT as persistence of the condition ≥ 6 months after surgery [11, 12]. In contrast, more recent recommendations, including the 2022 recommendations from the 2nd International Workshop and the recently 2025 European Society of Endocrinology Clinical Practice Guidelines defined chronic HPT when hypocalcaemia persist for more than 12 months after surgery [5, 13].

Data on the epidemiology of chronic HPT are sparse, as national and international disease-specific registries are still lacking. In Europe, the epidemiology of HPT has been investigated using non-anonymous, public health registries or public insurance claim databases. Based on systematic review and meta-analysis of published studies, the overall prevalence of HPT in Europe was estimated at 3.2 per 10,000 population in 2020 [14]. Based on this analysis the prevalence estimate for non-surgical HPT in the EU was 1.2/10,000 (95% CI : 0.6–1.6 per 10,000) and, the prevalence estimate for post-surgical HPT in the EU was 2.0/10,000 (95% CI : 1.6–2.3 per 10,000) [14]. In Denmark, data from regional/national registries reported a prevalence of 22/100,000 for postsurgical and 2.3/100,000 for non-surgical HPT, with a global prevalence of 25.4/100,000 [15, 16]. By contrast, studies relying solely on electronic hospital registries, such as those conducted in Norway, reported much lower prevalence estimates (5–10/100,000), with a relatively higher proportion of non-surgical cases [17].

In Italy, the mean prevalence of hypoparathyroidism among hospitalized patients was 5.3/100,000inhabitants per year in the period 2006–2013 was observed by the National Health Ministry database [18]. The prevalence of chronic HPT in a Mediterranean region was estimated at 27 per 100,000 inhabitants by using an indirect method based on analysis of active vitamin D metabolite (AVDM) prescriptions in Tuscany from 2009 to 2013 [19]. Marcucci and colleagues used data from 20 centers homogeneously distributed across Italy and reported that the leading etiology was represented by postsurgical HPT (67.6%) followed by idiopathic HPT (14.6%), syndromic forms of genetic HPT (11%), forms of defective PTH action (5.2%), non-syndromic forms of genetic HPT (0.9%) and other forms of acquired HPT (0.7%) [20].

Data on the prevalence and incidence of chronic HPT are highly variable, making it difficult to compare studies across different populations and clinical settings. The objective of this study was to review the existing literature and conduct a meta-analysis to provide a pooled estimate of chronic HPT among patients in Italy, thereby offering a clearer understanding of its prevalence in the Italian population.

## Materials and methods

### Data sources and search strategy

We conducted a systematic search of the literature published between 2010 and 2025 using PubMed and the Cochrane databases. The review protocol was registered and published in full on the Prospero website (protocol number: RD420251015275). This systematic review and meta-analysis were conducted in accordance with the Preferred Reporting Items for Systematic Reviews and Meta-Analyses (PRISMA) guidelines [21]. The following search terms were applied: “hypoparathyroidism AND epidemiology AND Italy”, “hypoparathyroidism AND incidence AND Italy”, “hypoparathyroidism AND prevalence AND Italy”, Hypocalcemia AND prevalence AND Italy”, “Hypocalcemia AND incidence AND Italy”, “Hypocalcemia AND epidemiology AND Italy”.

The number of thyroidectomies in Italy was searched for in Italian Ministry of Health Hospital Activity reports. In addition, a literature search was conducted in grey literature and PubMed (using the search string “thyroidectom*” AND “Italy”). The asterisk (*) denotes that the term was truncated and that all endings of the word were included in the search.

## Inclusion and exclusion criteria

Studies were included if they met all the following criteria: (1) reported data on the incidence or prevalence of HPT, referring to the Italian population (2) were published in English, (3) were performed in clinical settings such as hospitals, intensive care units, and emergency rooms, University hospitals. The following exclusion criteria were applied: (1) studies not in English, (2) case reports, review articles, editorials and opinions. We considered HPT as chronic when hypocalcaemia persists for more than 6 months’ duration for methodological consistency and comparability across the pooled data.

## Data extraction

Titles/abstracts screening, assessment for eligibility and data extraction were done by two review team members independently. The PRISMA flow chart was used to document the study selection. Titles and/or abstracts of studies retrieved using the search strategy and those from additional sources were screened to identify studies that potentially meet the inclusion criteria outlined above. The full text of these potentially eligible studies was retrieved and assessed for eligibility. A standardised, pre-piloted form was used to extract data from the included studies for assessment of study quality and evidence synthesis. Extracted information included: details of publication; geographical location; institution; type of study; study population; average age; proportion of sex; diagnostic criteria; prevalence and incidence data.

## Quality and bias assessment

The Checklist for Prevalence Studies from the Joanna Briggs Institute (JBI) Critical Appraisal tools for use in JBI Systematic Reviews was used to assess the methodological quality of each study and to determine the extent to which a study addressed the possibility of bias in its design, conduct and analysis [22]. All papers selected for inclusion in the systematic review were subjected to rigorous appraisal by two independent reviewers with disagreements settled by consensus. The quality appraisal did not aim to assign a score for inclusion/exclusion of papers, but to ascertain the extent to which the possibility of bias in the study design, conduct and analysis had been addressed. The results of the evaluation were incorporated in the synthesis and interpretation of the systematic review results. Papers with a score up to 49% reporting “yes” indicated a “high” risk of bias, 50–69% indicated a moderate risk of bias, and a score of 70% or higher reporting “yes” belonged to a low risk of bias.

## Data synthesis and analysis

We provided a narrative synthesis of the findings from the included studies. Given their definition, prevalence and incidence were reported as continuous variables (a proportion of cases in a predefined population). Hence, where studies have used the same type of outcome (prevalence or incidence), we pooled the results using a random-effects meta-analysis of proportion, thus reporting the aggregate incidence or prevalence, corresponding p-value, 95% confidence interval and estimated effect size (t²) of the outcome. Heterogeneity across studies was assessed using the I² statistic, which is commonly used to evaluate the degree of variation in effect sizes due to factors other than sampling error (Higgins et al., 2003). Additionally, forest plots were made for the prevalence and incidence of HPT and within subgroups of interest. All statistical analyses were performed using R (version 4.1.2, R Foundation for Statistical Computing, Vienna, Austria).

The prevalence rate for HPT per 100,000 population was calculated using the following formula: (duration of the disease x incidence cases/mean annual resident population) x 100,000 inhabitants, by considering the total number of inhabitants in the country, as indicated by the Italian National Statistics Institute database (available online at http://www.istat.it). The duration of the disease was calculated from the mean age at diagnosis to the mean age at death. The average age was derived from a meta-analysis of three Italian studies [20, 23, 24] and it was assumed that diagnosis of chronic HPT occurs on average one year after surgery. The HPT average mortality in Italy was calculated considering the average life expectancy in Italy as indicated by the Italian National Statistics Institute database (available online at http://www.istat.it) and a life-expectancy reduction for HPT patients [25].

## Results

**Fig. 1:**
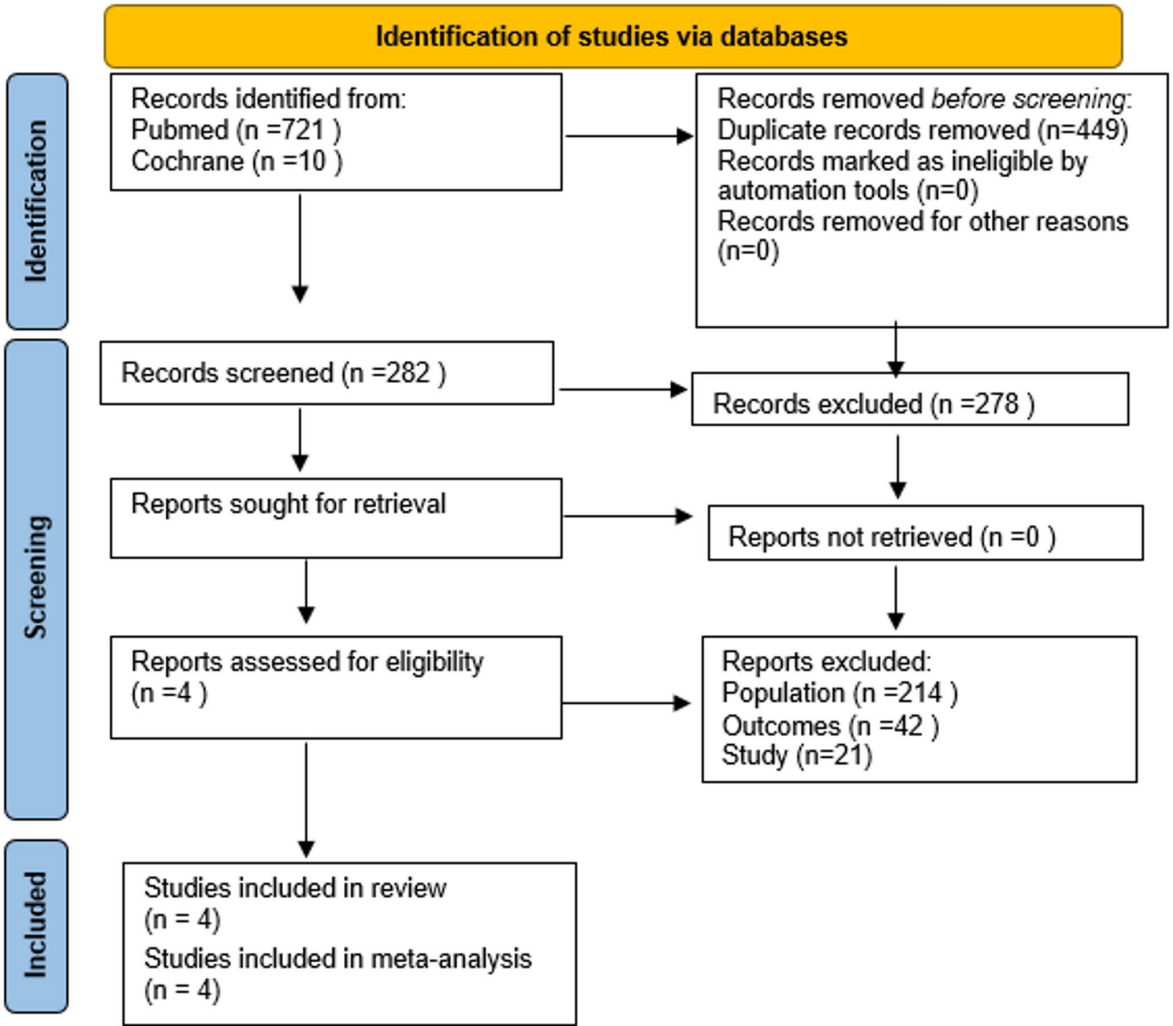
PRISMA flowchart

The PRISMA 2020 flow diagram for the study selection process is shown in Fig. 1. The literature search resulted in 731 articles published between 2010 and 2025. Based on the titles and abstracts, 282 articles were selected for the review. Finally, 4 papers were identified as eligible for inclusion in this systematic review and meta-analysis. According to the Joanna Briggs Institute Critical Appraisal Checklist, among 4 studies, one study had a moderate risk of bias, and 3 were studies with a low risk of bias (Table 1).

**Table 1:**
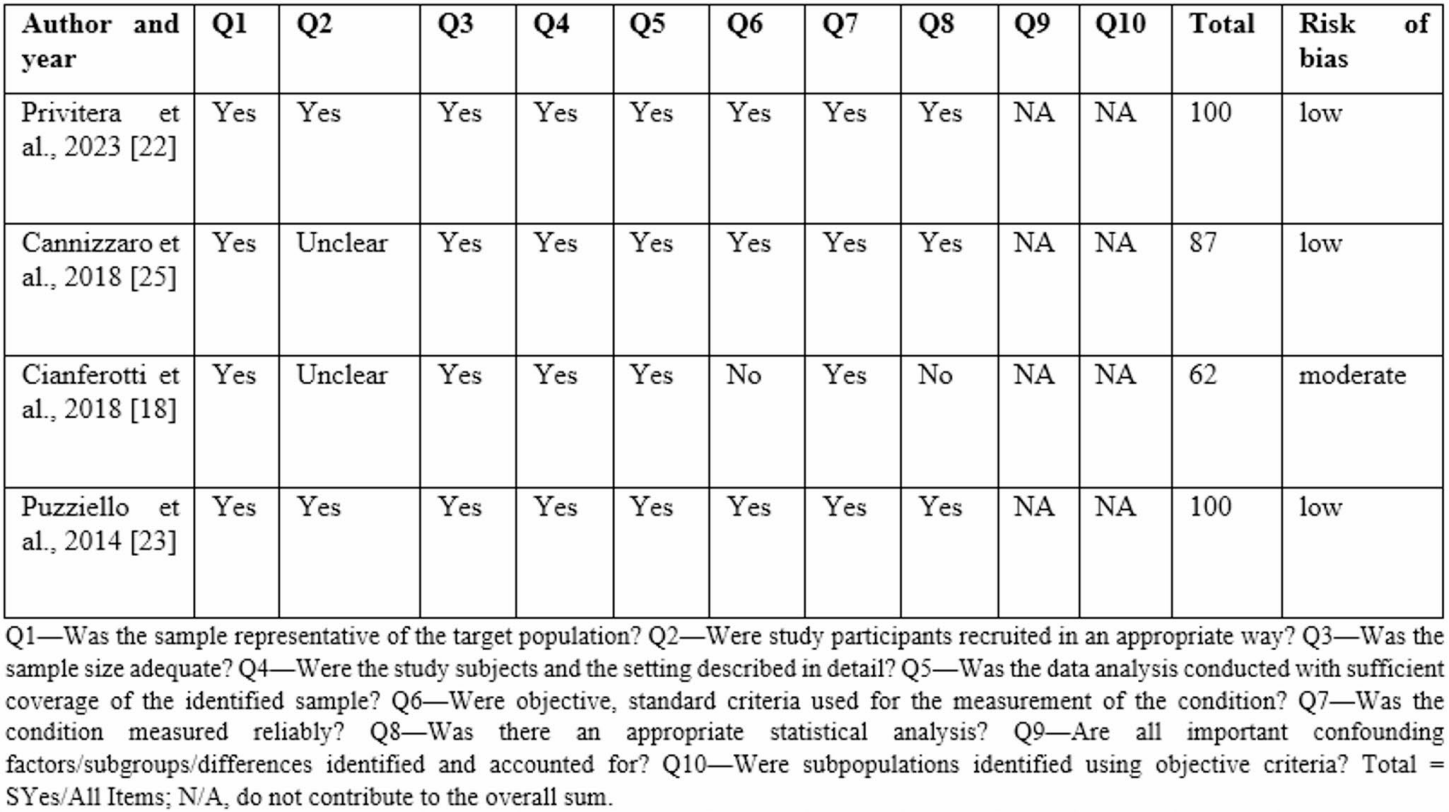
Results from the Joanna Briggs Institute critical appraisal checklist

Table 2 summarizes the characteristics of eligible studies. Data collection from Italian registries was retrospective in 3 studies and prospective in one study. Data were found across multiple settings at population level including hospitals (*n* = 2), surgery units (*n* = 1), electronic health records (*n* = 1). The four eligible studies reported cases of postoperative HPT, including 15,412 patients who underwent total or partial thyroidectomy, laryngectomy or thyroidectomy. The surgical indications were mostly benign thyroid disease also including recurrent benign goiter. The diagnosis of HPT was confirmed in 1,363 patients based on laboratory parameters in combination with hypocalcemia, which was defined as a serum calcium concentration < 8.0 mg/dL, ionized calcium < 1.12 mmol/L, or the use of active vitamin D metabolites (AVDM) as treatment. In most of cases hypocalcemia was transient (< 6 months after surgery) and, 0.6–2.3% of patients included in the analysis experienced chronic hypocalcemia.

**Table 2:**
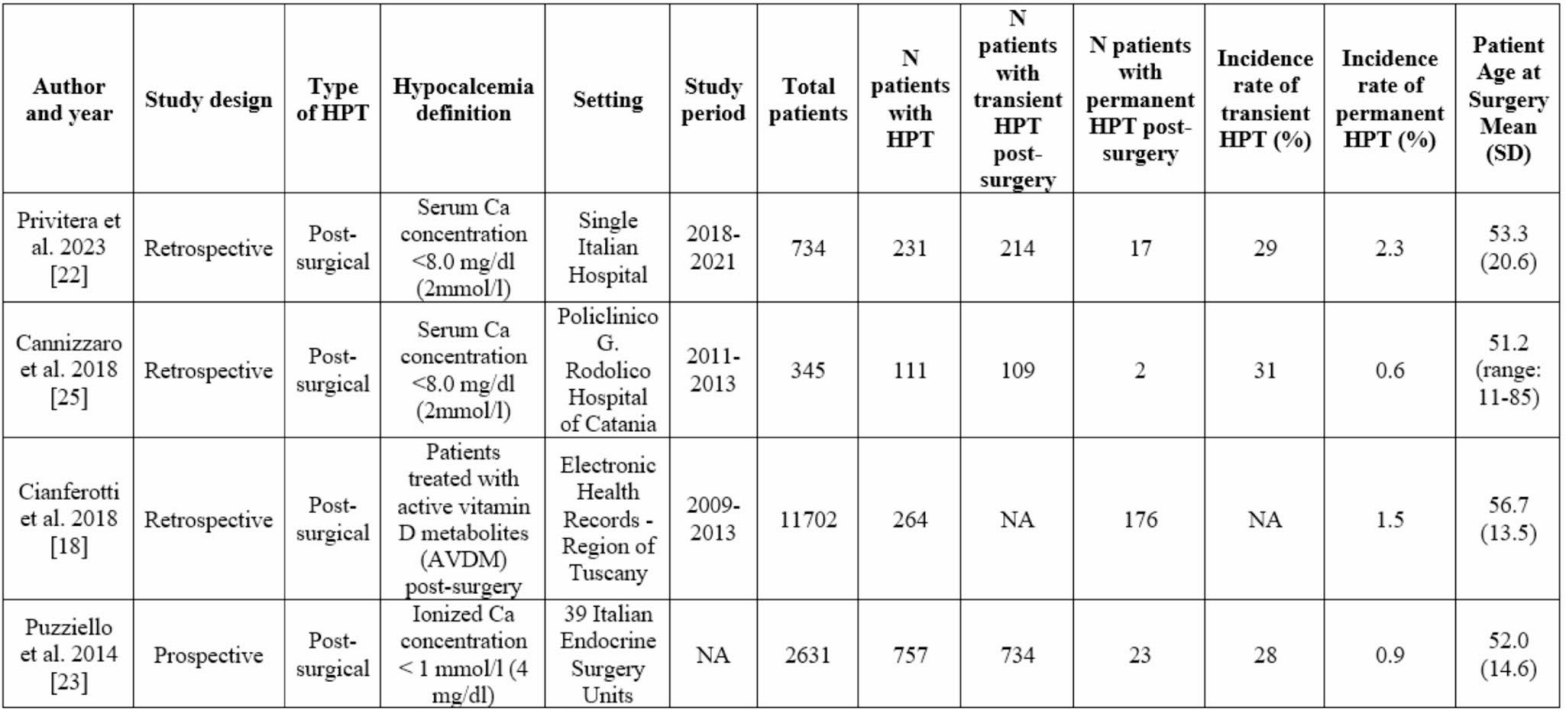
Characteristics of included studies

## Incidence of post-surgical HPT

**Fig. 2:**
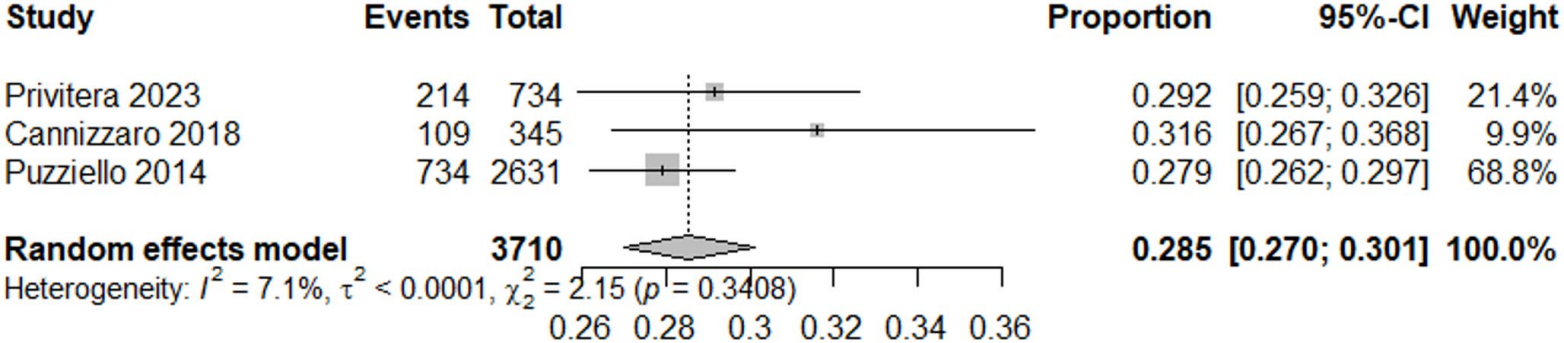
Meta-analysis of the incidence of transient post-surgical HPT in Italy

**Fig. 3:**
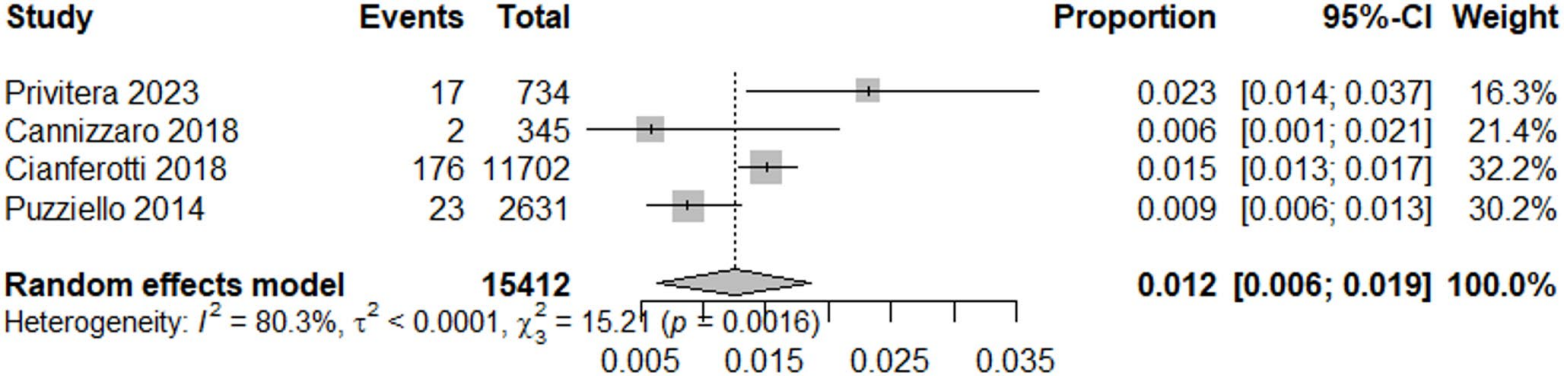
Meta-analysis of the incidence of chronic post-surgical HPT in Italy

The selected studies reported incidence of post-surgical HPT in Italy and were analyzed via a random-effects meta-analysis. The estimated pooled incidence of transient HPT following surgery was 28% (95% CI: 27%–30%; I² = 7.1%) (Fig. 2). This estimate was derived from three studies that provided data on the number of transient HPT cases [23, 24, 26]. The study by Cianferotti et al. [19] was not included, as it did not report the number of transient HPT cases. The pooled incidence of chronic HPT (≥ 6 months after surgery) was 1.2% (95% CI: 0,7%−1,8%; I^2^ = 80,3%) (Fig. 3).

**Fig. 4:**
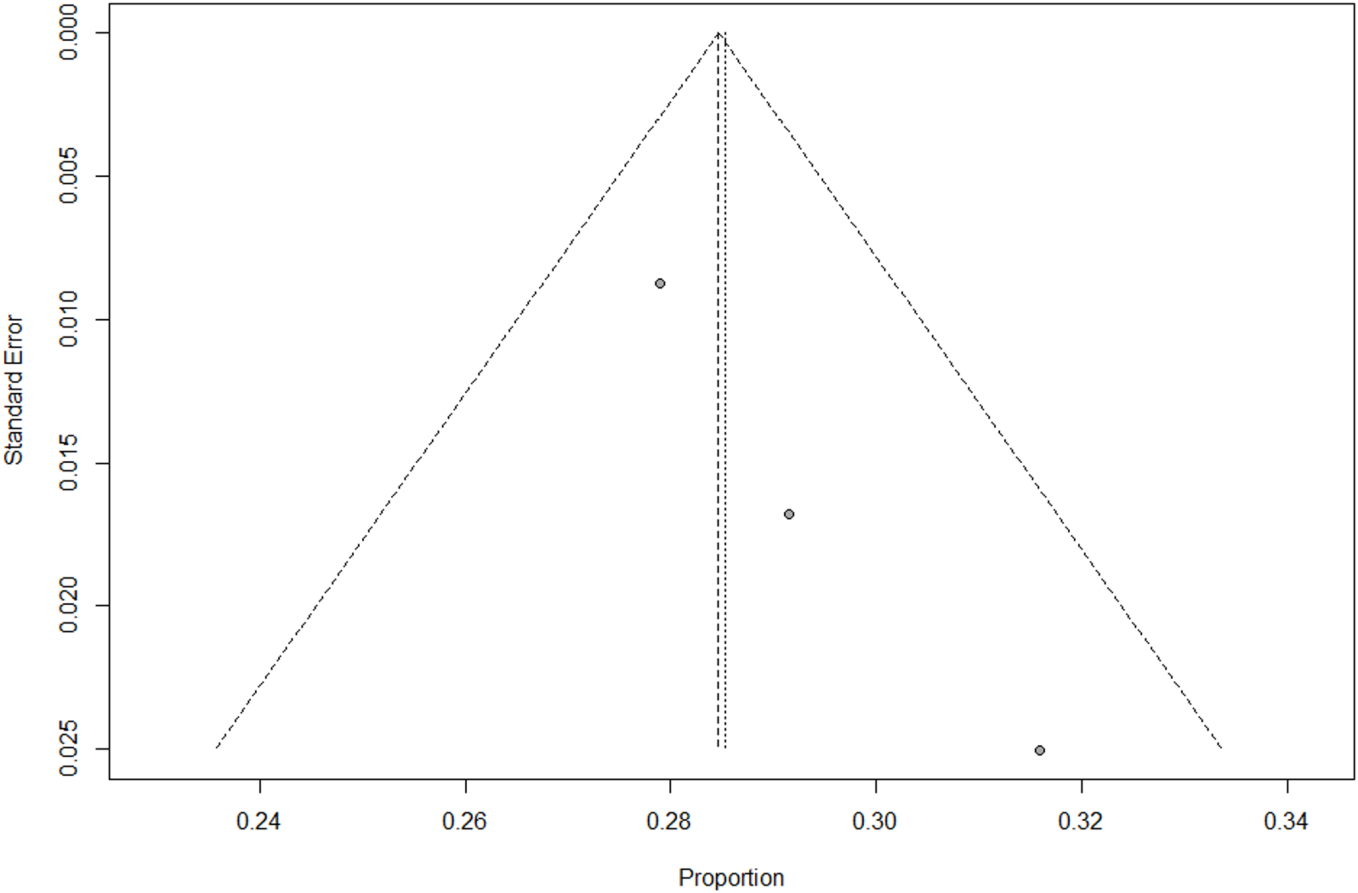
Funnel plot for incidence of transient HPT. Note: dots represent individual studies; the outer dotted line indicates the triangular region within which 95% of studies are expected to lie in the absence of both biases and heterogeneity; the solid vertical line represents the line of no effect, derived using fixed-effect meta-analysis

**Fig. 5:**
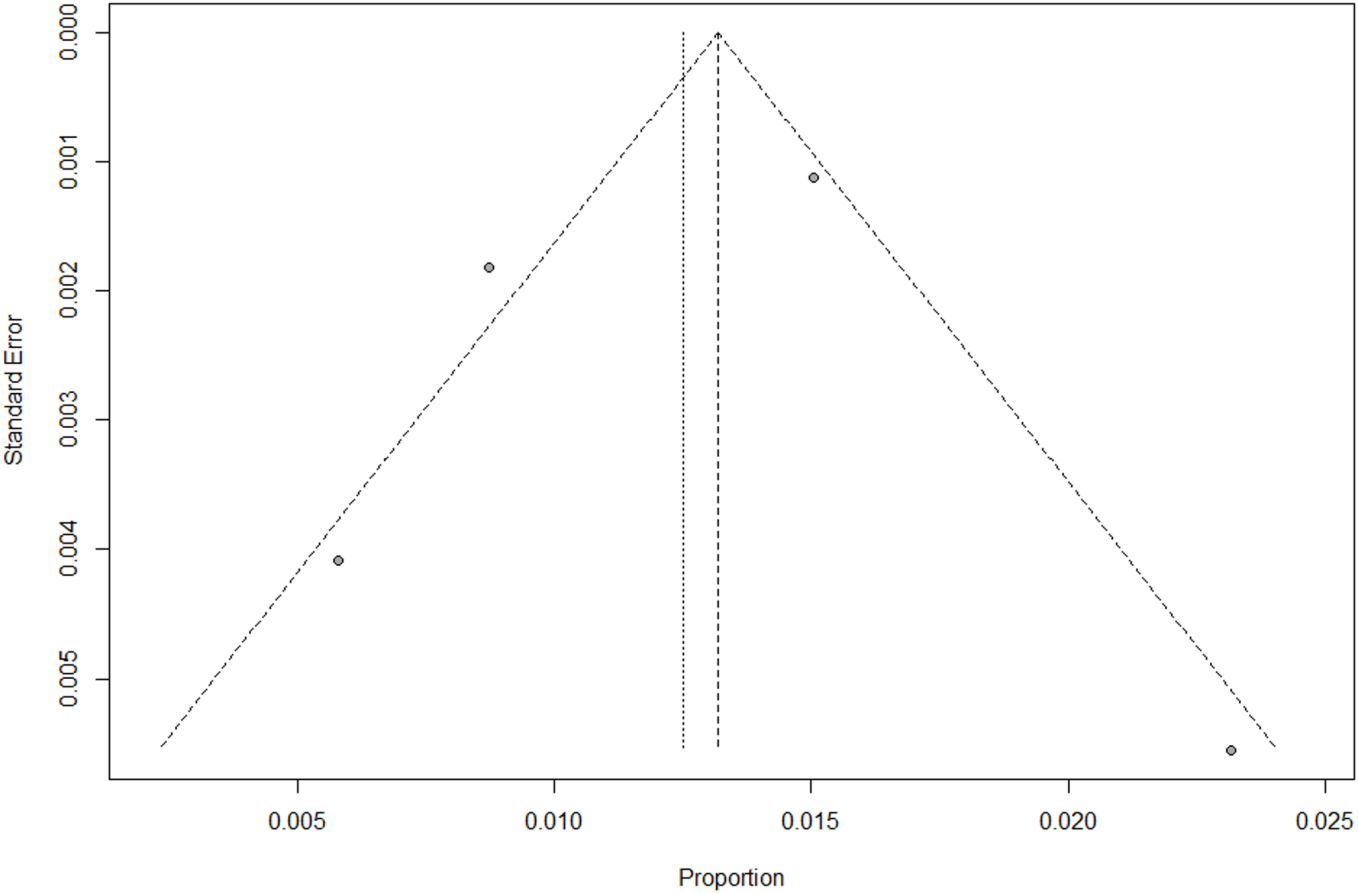
Funnel plot for incidence of chronic HPT. Note: dots represent individual studies; the outer dotted line indicates the triangular region within which 95% of studies are expected to lie in the absence of both biases and heterogeneity; the solid vertical line represents the line of no effect, derived using fixed-effect meta-analysis

Publication bias of selected studies was assessed by funnel plots separately for incidence of transient and chronic HPT (Figs. 4 and 5). Egger’s test results indicated no publication bias. However, this meta-analysis contains k = 4 studies. Egger’s test may lack the statistical power to detect bias when the number of studies is small (i.e., k < 10).

During the 2001–2018 period a total number of 704,523 thyroidectomies were performed in Italy, corresponding to ~ 40,000 per year [27]. Thyroidectomy rates decreased over time from nearly 60 per 100,000 in 2012 to 48 per 100,000 in 2018 [27]. The trend of reduction in thyroidectomy rates was also confirmed by recent data published in the Italian Ministry of Health 2021 Hospital Activity Report, reporting a thyroidectomy rate of approximately 57 per 100,000 in 2021 [28].

Considering Italy’s current adult population of 50,170,040 (ISTAT, 2025) and a thyroidectomy rate of 57 per 100,000, this translates to 28,425 thyroidectomies in 2025. Applying the post-surgical chronic HPT incidence rate (1.2%) to the total number of thyroidectomies in Italy (28,425), the number of incident post-surgical chronic HPT cases was estimated at 353 cases in 2025.

## Prevalence of HPT in Italy

**Fig. 6:**
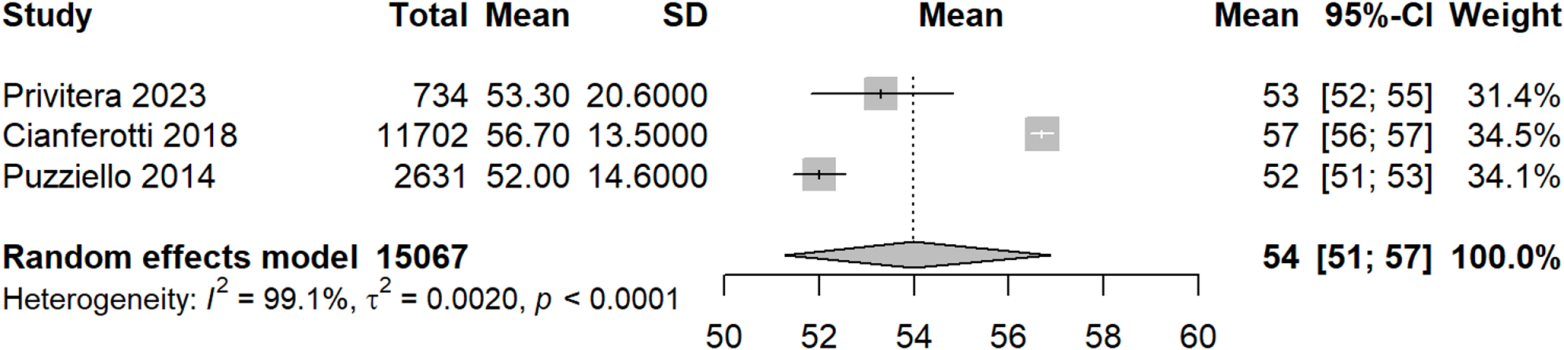
Meta analysis-mean age of thyroid surgery in Italy

The annual incidence of postsurgical chronic HPT was translated into prevalence using duration of disease, which was calculated from mean age at diagnosis through mean age at death. The average age for thyroid surgery (54 years) was derived from meta-analysis of three studies [19, 23, 24] (Fig. 6). The study by Cannizzaro et al. [26] was excluded from the analysis because it did not provide the standard deviation of the outcome measures. As the standard deviation is required to quantify the variability and to calculate effect size estimates, the absence of this information made it impossible to include the study in the quantitative analysis. It was assumed that diagnosis of chronic HPT occurs on average one year after surgery. The HPT average mortality in Italy was calculated considering the average life expectancy in Italy (83.4 years) (ISTAT 2025) and an 11-year life-expectancy reduction for HPT patients [26], which lead to a mean age at death of 72.4 years and thus a mean time with chronic HPT after diagnosis of 17.4 years. The prevalence of postsurgical chronic HPT was estimated to be 6,142 cases for Italy (Table 2).

The prevalence of non-surgical HPT was estimated from a study published by Marcucci 2018 [20]; that estimated 32.4% of chronic HPT cases with non-surgical etiology. This percentage was then applied to Cianferotti’s total prevalence estimate [19] and incorporated into the meta-analysis calculation yielding a non-surgical prevalence rate of 8.7 per 100,000 individuals. Findings from this analysis were adjusted to account for Italy’s population demographics, resulting in 4,383 prevalent non-surgical chronic HPT cases in Italy. The prevalent adult population with chronic HPT in Italy, both surgical and non-surgical, was estimated to be 10,524 cases (Table 3).

**Table 3:**
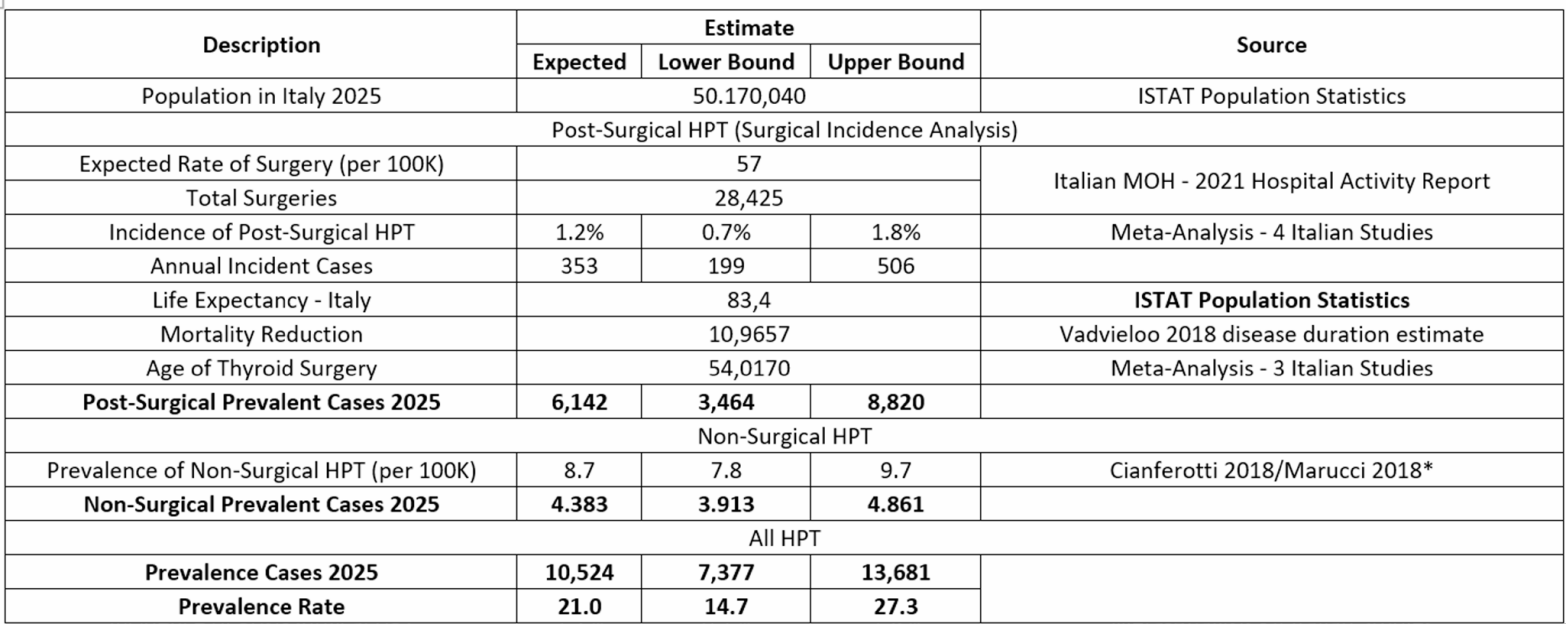
HPT estimates 2025 – Italy

## Discussion

This study represents the first systematic review and meta-analysis examining the epidemiology of HPT in Italy. We provided national estimates for the incidence and prevalence of the disease, identifying approximately 353 incident cases of postsurgical chronic HPT and projecting a total of 10,524 prevalent cases of chronic HPT (both surgical and non-surgical) in the adult population in 2025.

Our analysis highlights that postsurgical HPT is the most common etiology in Italy, consistent with patterns observed in other countries. The variability in incidence and prevalence of HPT appears primarily driven by surgical practices, particularly the type of neck surgeries performed, as well as by the criteria used to define and diagnose HPT. Importantly, the incidence of postsurgical HPT is influenced not only by surgical technique, but also by surgeon experience and institutional surgical volume, both of which are well-recognized determinants of postoperative outcomes in thyroid and parathyroid surgery. High-volume surgeons and centers have consistently been associated with lower rates of permanent hypoparathyroidism [29]. Reported estimated rates of postsurgical HPT in the literature range from 1.7% to 68%, influenced by underlying pathology, surgical technique, perioperative management, and healthcare setting [30–33].

There is also a notable inconsistency in how HPT is defined across studies. Parameters used in clinical studies to define transient postsurgical HPT include serum calcium levels alone or calcium levels and symptoms of HPT; many studies employed the 6-month term after surgery to define chronic HPT [34].

Our observation of a 1.2% (95% CI: 0,7%−1,8%) incidence rate of chronic post-surgical HPT, defined as lasting ≥ 6 months after surgery, is in line with previous global systematic review and meta-analysis of 115 studies, reporting an incidence of transient and chronic post-surgical hypocalcaemia of 27% (19–38%) and 1% (0–3%), respectively [35].

More recently, a decreased risk of chronic HPT after neck surgery was observed by clinical studies, in accordance with an increased surgical expertise; conversely, rare forms once defined as idiopathic are now more often recognized and genetically diagnosed because of improvements in tools used for molecular diagnosis [1].

Italian registry data further support our findings. A retrospective analysis involving 20 Italian endocrinology and endocrine surgery centers identified 537 patients with chronic HPT, with 67.6% of cases attributed to surgery, followed by idiopathic (14.6%) and genetic (11%) causes [20]. Interestingly, a decline in HPT incidence was observed, coinciding with a ~ 23% reduction in thyroidectomy rates during the same period [28, 29].

Previous Italian epidemiological data have estimated HPT prevalence at 5.3 per 100,000 individuals annually based on hospital discharge records [18]. However, such estimates likely underestimate the true prevalence due to the exclusion of non-hospitalized patients [18]. An indirect and more inclusive approach by Cianferotti et al., using prescription data for active vitamin D metabolites in Tuscany, found a prevalence of 27 per 100,000 [19], which closely aligns with our own findings, validating the reliability of our estimates.

Population‑based analyses indicated that prevalence of post‑surgical chronic HPT in Europe is relatively low but variable depending on methodology and definition. A systematic evaluation of European studies estimated the prevalence of post‑surgical hypoparathyroidism at approximately 2.0 per 10,000 individuals (95% CI: 1.6–2.3 per 10,000) as part of an overall hypoparathyroidism prevalence of 3.2 per 10,000 in the EU (with separation of surgical and non‑surgical cases) [15]. National registry and database studies from Denmark reported a prevalence of 22 per 100,000 person‑years for hypoparathyroidism, with surgical causes predominating over non‑surgical ones [10]. Data of regional prescription database from northern Spain have identified a chronic post‑surgical hypoparathyroidism prevalence of approximately 31.7 per 100,000 inhabitants [36]. Although direct comparisons are limited by differences in study design, follow‑up duration, and criteria for defining chronic disease, these figures suggest that our Italian estimates fall within the broader range observed across European settings. Variability in healthcare systems, surgical expertise, and diagnostic practices likely contribute to the observed differences in prevalence and incidence.

A potential limitation of our study is related to the definition of chronic HPT. In our analysis, chronic HPT was defined as persistence of hypocalcaemia for ≥ 6 months after surgery, which is consistent with the majority of published studies. However, more recent guidelines [13], including the 2022 International Workshop [5], recommended a ≥ 12-month threshold to define chronic HPT. As a result, the use of a ≥ 6-month definition may slightly overestimate the prevalence of chronic HPT compared with recent criteria.

Another limitation of our meta-analysis is the limited number of eligible studies and substantial heterogeneity that was observed across these studies, related to differences in study design, publication year, surgical expertise and clinical setting. Additionally, the estimates rely on modelling assumptions, which may not fully capture variability in patient populations and clinical practices. It is advised to interpret these findings with caution, recognizing that further studies are needed to improve the robustness of national-level estimates.

In conclusion, we provide an estimate of the prevalence and annual incidence rate of chronic HPT in Italy, as derived from the meta-analysis of the most relevant and recent epidemiological studies published in the field. The chronic postsurgical HPT represents the most common etiology. Future epidemiological studies with appropriate study design are warranted to provide a validated estimation of the actual incidence and prevalence of chronic HPT in Italy.
